# Herpesvirus Diversity in Stranded Mysticetes of Brazil, Southwestern Atlantic

**DOI:** 10.3390/v18060612

**Published:** 2026-05-27

**Authors:** Vanessa Dal Bianco, José Luiz Catão-Dias, Roberta Zamana-Ramblas, Samira Costa-Silva, Ana Carolina Ewbank, Barbara Sophia Codeas, Adriana Castaldo Colosio, Marta J. Cremer, Jenyffer V. Vieira, Giulia G. Lemos, Carlos Sacristán, Aricia Duarte-Benvenuto

**Affiliations:** 1 Faculdade de Medicina Veterinária e Zootecnia, Universidade de São Paulo, São Paulo 05508-270, SP, Brazil; vanessabianco@usp.br (V.D.B.); zecatao@usp.br (J.L.C.-D.); rrzamana@gmail.com (R.Z.-R.); barbara.sophia@alumni.usp.br (B.S.C.); 2 Instituto Baleia Jubarte, Caravelas 45900-000, BA, Brazil; costasilva.samira@gmail.com (S.C.-S.);; 3 Centro de Investigación en Sanidad Animal (CISA-INIA), CSIC, 28130 Valdeolmos, Madrid, Spain; ewbank@inia.csic.es; 4 Laboratório de Ecologia e Conservação de Tetrápodes Marinhos e Costeiros—TETRAMAR, Universidade da Região de Joinville, Joinville 89240-000, SC, Brazil; mjc2209@yahoo.com.br (M.J.C.); jenyffer.vieira@univille.br (J.V.V.); giulia.lemos@univille.br (G.G.L.)

**Keywords:** emerging infectious diseases, marine mammal, molecular epidemiology, mysticetes, Bryde’s whale, common minke whale, humpback whale

## Abstract

Emerging and re-emerging pathogens have been increasingly investigated in cetaceans worldwide, particularly in odontocetes, whereas infectious diseases in mysticetes remain poorly understood. This study surveyed herpesvirus in 51 baleen whales stranded along the Brazilian coast, between 2008 and 2022: humpback whales (*Megaptera novaeangliae*, n = 46), Bryde’s whales (*Balaenoptera brydei*, n = 3), a southern right whale (*Eubalaena australis*, n = 1), and a common minke whale (*Balaenoptera acutorostrata*, n = 1). A total of 159 tissue samples were analyzed using nested PCR assays targeting the herpesviral DNA polymerase (DPOL) and glycoprotein B (gB) genes. Herpesvirus DNA was detected in 25.5% (13/51) of individuals, including 23.9% (11/46) of humpback whales, one Bryde’s whale, and one common minke whale. Positive animals included six calves, six juveniles, and one adult. All 13 cases were detected by DPOL PCR, yielding eight sequence types (five alphaherpesviruses and three gammaherpesviruses). Additionally, six of them were also positive for gB, identifying four gammaherpesvirus sequence types within the genus *Rhadinovirus*. Humpback whales showed both alpha- and gammaherpesviruses, including one coinfection, whereas gammaherpesviruses were detected in Bryde’s and minke whales. This study reports the first herpesvirus in Bryde’s whales and expands the geographic range of infection in minke whales. The high prevalence in calves suggests vertical transmission.

## 1. Introduction

The order Cetacea comprises two suborders: Odontoceti (toothed whales) and Mysticeti (baleen whales), which differ primarily in feeding strategies, morphology, and ecological roles [1]. Odontocetes possess teeth and use echolocation for prey detection, while mysticetes are filter-feeding mammals and have baleen plates [1]. Cetaceans are considered sentinel species of the aquatic environment reflecting the presence of environmental pollutants and pathogens due to their position in the food chain, longevity, and lipid-rich blubber that bioaccumulates lipophilic contaminants [2,3,4]. In Brazil, the two main mysticete species that seasonally migrate to its territorial waters for reproduction are the southern right whale (*Eubalaena australis*), which concentrates on the southern coast [5], and the humpback whale (*Megaptera novaeangliae*), whose reproductive range extends from southeastern to northeastern Brazil, primarily around the Abrolhos Archipelago [6]. In the 20th century, both species experienced a sharp population decline due to anthropogenic actions, especially commercial whaling. After the ban on whaling in 1985 [7], habitat degradation, environmental pollution, and vessel collisions became the main threats to the conservation of these animals [8]. Several emerging and re-emerging pathogens are increasingly reported in cetaceans worldwide, mainly in odontocetes [9,10,11,12]. Nevertheless, knowledge of infectious diseases and causes of mortality in mysticetes remains limited [9,10,13,14,15].

Herpesviruses are double-stranded enveloped DNA viruses classified into three families: *Malacoherpesviridae* (infecting mollusks), *Alloherpesviridae* (in fish and amphibians), and *Orthoherpesviridae* (in reptiles, birds, and mammals) [16]. The latter family is further subdivided into the subfamilies *Alphaherpesvirinae*, *Betaherpesvirinae*, and *Gammaherpesvirinae* [16,17,18]. The relationship between herpesviruses and their hosts is usually species-specific, characterized by latency in natural hosts and the development of lesions under immunosuppression or in non-adapted species [16,19]. In odontocetes, alphaherpesvirus infections have been described in association with cutaneous and mucocutaneous lesions, encephalitis, vasculitis and nephritis, with local or systemic involvement, while gammaherpesviruses are mainly associated with cutaneous and mucosal lesions [17,18,20,21]. Nevertheless, both subfamilies were also reported in individuals without lesions [17,18,20,21]. In mysticetes, these viruses have been detected exclusively in the family Balaenopteridae, specifically in fin whales (*Balaenoptera physalus*), common minke whales (*Balaenoptera acutorostrata*) and humpback whales [22,23,24,25]. To date, there are only seven published reports of herpesvirus infections in mysticetes. Alphaherpesviruses have been described in the skin and penile mucosa of a fin whale in the Mediterranean coast of Spain [22], in the lung of a humpback whale stranded on the Brazilian coast [24], in a skin lesion in a humpback whale from the Spanish Mediterranean Sea [25], in pooled blow samples of humpback whales of Japan [26], and in blow samples of humpback whales of Iceland, Norway and Cape Verde [27]. Gammaherpesviruses have been detected in the skin, muscle, and central nervous system of a common minke whale stranded on the Mediterranean coast of Spain [22], in the central nervous system of another common minke whale in the UK [23], and in the skin biopsy of a humpback whale of Norway [27]. Overall, the diversity, pathogenicity, and transmission of herpesviruses in mysticetes remain poorly understood.

This study aimed to investigate the herpesvirus occurrence in mysticetes stranded along the Brazilian coast to improve understanding of their epidemiology and potential conservation implications.

## 2. Materials and Methods

### 2.1. Samples

A total of 51 dead stranded mysticetes were analyzed in this study, comprising four species: humpback whale (n = 46), Bryde’s whale (*Balaenoptera brydei*, n = 3), common minke whale (*Balaenoptera acutorostrata*, n = 1), and southern right whale (n = 1). Individuals were selected based on their decomposition status, which was assessed according to external and internal carcass condition and categorized using standard codes ranging from Code 1 (fresh: live or recently deceased) to Code 5 (advanced decomposition), corresponding to fresh, mild, moderate, and advanced decomposition stages, with preference given to Code 1–3 specimens [28]. The age class (calf, juvenile, and adult) was estimated according to Lodi and Borobia [1] and Christiansen et al. [29], based on the overall body size and the degree of morphological development. Based on these criteria, 30 individuals were classified as calves, 14 as juveniles, six as adults, and one as undetermined age. Regarding sex, 28 were males, 21 were females, and two were undetermined.

All animals stranded on the coast of the northeastern (n = 28), southeastern (n = 13), and southern (n = 10) regions of Brazil, between 2008 and 2022. Necropsies were performed by veterinarians from the Instituto Baleia Jubarte (Bahía and Espirito Santo states) and the Laboratory of Ecology and Conservation of Marine and Coastal Tetrapods (TETRAMAR) at the University of the Region of Joinville (Santa Catarina state), following a standardized protocol [28]. Tissue samples from multiple organs (i.e., bladder, brain, heart, lymph nodes, skin, lung, and kidney) were systematically collected from each individual, preserved in 10% buffered formalin for histopathological analyses and frozen (−20 °C, −80 °C) for molecular analyses.

### 2.2. Molecular Analyses

An aliquot of up to 3 mm^3^ was collected from each available frozen tissue per individual (i.e., bladder, brain, heart, lymph nodes, skin, lung, and kidney), and each aliquot was individually homogenized using 180 μL of buffer ATL, four beads and the Bead Ruptor 12 (OMNI international, NW Kennesaw, GA, USA). Overall, total DNA was extracted from 159 samples using the DNeasy Blood & Tissue Kit (Qiagen^®^, Valencia, CA, USA), according to the manufacturer’s instructions. Extracted DNA was frozen at −20 °C until processing.

The total DNA of each tissue sample was individually tested for herpesvirus using a broad-spectrum nested PCR that partially amplifies a conserved region of the DNA polymerase (DPOL) gene from a wide variety of species from the *Alphaherpesvirinae*, *Betaherpesvirinae*, and *Gammaherpesvirinae* subfamilies [30]. The DPOL assay employed degenerate primers in two amplification rounds of 40 cycles each, with an annealing temperature of 56 °C, generating amplicons of approximately 215–315 bp. Additionally, a nested PCR specific for partial amplification of the glycoprotein B (gB) gene from the *Gammaherpesvirinae* subfamily was performed [31], employing the degenerate primer set GH1s in a nested format with 45 cycles each round, an annealing temperature of 46 °C and generating amplicon of approximately 500 bp. Appropriate positive and negative controls were included in all PCRs. Amplified fragments were visualized by 1.5% agarose gel electrophoresis, stained with SYBR Safe (Invitrogen, Carlsbad, CA, USA). Amplicons of the expected size were purified with ExoSAP-IT one-step enzymatic PCR product clean-up reagent (Affymetrix, Santa Clara, CA, USA) and directly sequenced in both directions by Sanger.

The obtained consensus sequences were compared with the closest matches available in the GenBank/DDBJ/ENA databases, using the BLAST (BLAST+ 2.17.0) tool (https://blast.ncbi.nlm.nih.gov/Blast.cgi). Sequences recovered in this study were aligned with the most similar ones in the international databases, and the percentage of similarity between them was measured by calculating the p-distance using the MEGA11 [32] software. The gB and the two DPOL phylograms (one for alphaherpesviruses and one for the gammaherpesviruses) were inferred from 1000 replicates and constructed using MEGA11. The evolutionary models were selected using ProtTest [33].

### 2.3. Gross Examination and Histopathology

Necropsy records of herpesvirus-positive animals identified by molecular diagnosis and classified within decomposition Codes 1–3 (fresh to mild decomposition) were reviewed, and gross findings were described. Tissue samples (i.e., bladder, brain, heart, lymph nodes, skin, lung, and kidney) were collected during necropsy and fixed in 10% neutral buffered formalin, processed, embedded in paraffin wax, sectioned at a 4 µm thickness using a rotary microtome, and stained with hematoxylin and eosin for histopathological analysis.

## 3. Results

### 3.1. Molecular Herpesvirus Detection

The overall herpesvirus prevalence in this study was 25.5% (13/51): 23.9% (11/46) of the humpback whales, one of the three Bryde’s whales and the common minke whale, whereas the southern right whale was herpesvirus-negative. Regarding age class, six out of 30 (20%) calves, six out of 14 (42.9%) juveniles, and one out of six (16.6%) adults tested positive. One individual of undetermined age tested negative. In regard to sex, seven out of 21 (33.3%) females and six out of 28 (17%) males tested positive, while two other individuals of unknown sex were negative. Detailed biological and epidemiological data of the herpesvirus-positive individuals are described in Table 1. Information for all tested individuals is available in Table S1.

Herpesvirus DNA was detected in various tissue samples, including lymph nodes (45%; 5/11), skin (9%; 2/21), lungs (15%; 5/33), kidney (14%; 5/36), brain (9%; 1/11), bladder (5%; 1/19), and heart (3%; 1/28) (Table 2).

### 3.2. Identity Analyses and Phylogeny

Seven whales were positive only for the DPOL protocol, while six tested positive for both DPOL and gB protocols. Overall, 13 animals were positive for the DPOL protocol (i.e., 11 humpback whales, one Bryde’s whale and the common minke whale), corresponding to eight nucleotide (nt) sequence types (STs): (i) five STs were classified as alphaherpesvirus, retrieved from five humpback whales; and (ii) three STs as gammaherpesvirus, found in a Bryde’s whale, a common minke whale and in seven humpback whales (one of them confected by alpha), respectively.

In the gB protocol, four nt STs of gammaherpesvirus were obtained from five individuals (i.e., four humpback whales and one common minke whale), while another humpback whale was gammaherpesvirus-positive, although sequence quality was insufficient for ST analyses.

In summary, a Bryde’s whale, a common minke whale and six humpback whales were positive for gammaherpesvirus, four humpback whales were positive for alphaherpesvirus, and one humpback whale (Mn1608) was co-infected with both. Five out of the 13 positive mysticetes showed positivity in more than one tissue. The molecular results of herpesvirus-positive whales and tissues are described in Table 2.

The alphaherpesvirus DPOL STs detected in humpback whales in this study showed nt identities ranging from 93.6% to 99.4% among them, and amino acid (aa) identities ranging from 91.2% to 100%. One of these alphaherpesviruses DPOL STs (Mn 1608) showed nt and aa identities of 95.3% and 97.2% to an alphaherpesvirus sequence obtained from a humpback whale from Norway (OQ561785), respectively. Two other STs (Mn 1642, Mn 1643) had nt sequences identical and highly similar (99.4%), respectively, to an alphaherpesvirus sequence described in another humpback whale from Norway (PP857845), while the remaining two (Mn 1617, Mn 1619) had the highest nt similarity (99.4% to 100%) to sequences detected in humpback whales of Brazil (OQ533671), Iceland (PP857848), Cape Verde (PP857846) and Norway (PP857844). The four latter STs showed aa identities ranging from 98.2% to 100% to a sequence detected in humpback whales from Brazil (OQ533671), Iceland (PP857848), Cape Verde (PP857846), and Norway (PP857843, PP857844, PP857845).

The gammaherpesvirus DPOL STs detected in the Bryde’s whale, in the minke whale, and in seven humpback whales, showed the highest similarities, to a common minke whale sequence from Spain deposited at GenBank/DDBJ/ENA under accession number KP995688, with nt and aa identities of 99.4% and 100%, 95.8% and 94.5%, and 81.4% and 85.5%, respectively.

Three nt gB STs were obtained in humpback whales: one in case Mn785, one in case Mn1054, and another one in cases Mn1081 and Mn1608. When translated into aa, the gB sequences from Mn785, Mn1081 and Mn1608 were identical. These sequences showed nt identities ranging from 99.8% to 100% among themselves, and aa identities ranging from 99.3% to 100%. Additionally, the nt and aa identities of the gB herpesvirus sequences obtained in humpback whales varied between 79.2–79.7% and 84.5–85.2%, respectively, compared to the most similar sequences available, detected in Atlantic spotted dolphins (*Stenella frontalis*, OQ926585, OQ926586) and Bolivian river dolphin (*Inia boliviensis*, MZ209285) of Brazil, respectively. The gB sequence amplified from a common minke whale showed nt and aa identities of 81.3% and 84.5% to sequences detected in Atlantic spotted dolphins (OQ926585, OQ926586), respectively.

**Figure 1:**
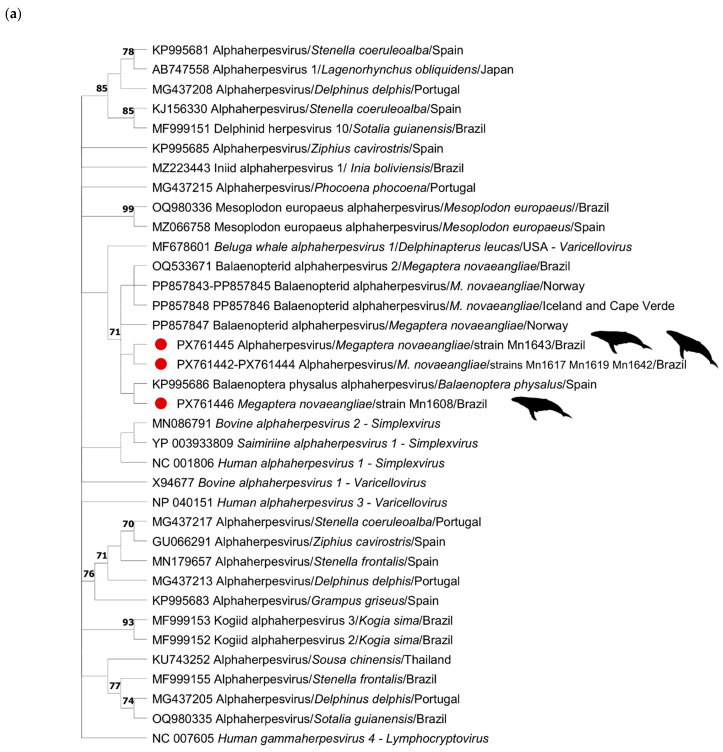

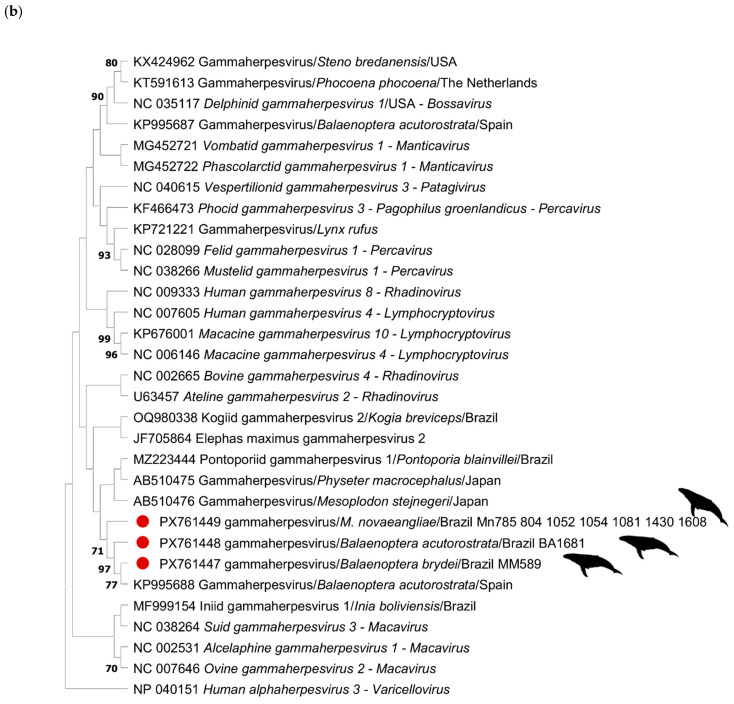

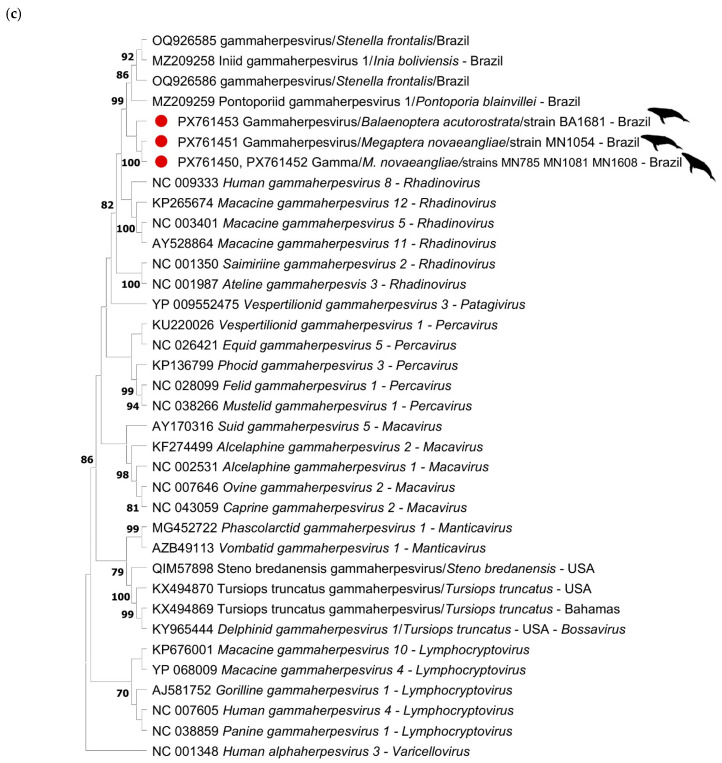
Consensus maximum likelihood phylogenetic trees of the amino acid alignment of: (**a**) DNA polymerase alphaherpesvirus sequences obtained in whales from Brazil in this study (identified with red dots and whale silhouettes), other alphaherpesvirus sequences from cetaceans, selected alphaherpesviruses within the *Simplexvirus* and *Varicellovirus* genera and *Human gammaherpesvirus 4* as an outgroup; (**b**) DNA polymerase gammaherpesvirus sequences obtained from whales from Brazil in this study (identified with red dots and whale silhouettes), gammaherpesviruses sequences previously detected in cetaceans, selected gammaherpesvirus species within the genera *Bossavirus*, *Lymphocryptovirus*, *Macavirus*, *Manticavirus*, *Patagivirus*, *Rhadinovirus*, and *Human alphaherpesvirus 3* as an outgroup; and (**c**) glycoprotein B gammaherpesvirus sequences obtained in whales from Brazil in this study (identified with red dots and whale silhouettes), gammaherpesviruses sequences previously detected in cetaceans, selected gammaherpesvirus species within the genera *Bossavirus*, *Lymphocryptovirus*, *Macavirus*, *Manticavirus*, *Patagivirus*, *Rhadinovirus*, and *Human alphaherpesvirus 3* as an outgroup. The evolutionary models used were Jones–Taylor–Thornton model with gamma-distributed rate variation among sites for the DNA polymerase alphaherpesvirus tree and the Le and Gascuel model with gamma-distributed rate variation among sites for the DNA polymerase and glycoprotein B gammaherpesvirus trees. The trees were generated with 1000 bootstrap replicates. Bootstrap values below 70 were omitted; bootstrap values equal to or higher than 70 are presented in bold.

All unique deduced DPOL aa sequences of alphaherpesviruses obtained from mysticetes clustered together with a bootstrap support of 71%, forming a distinct lineage that was not classified within the genera *Simplexvirus* or *Varicellovirus* (Figure 1a). The deduced DPOL aa STs of the gammaherpesviruses detected in a Bryde’s whale, a common minke whale, and seven humpback whales clustered with gammaherpesvirus previously identified in a common minke whale from Spain (Figure 1b).

The gammaherpesvirus gB aa sequences from a common minke whale and four humpback whales clustered within the genus *Rhadinovirus*, together with gammaherpesvirus sequences obtained from Atlantic spotted dolphin (*Stenella frontalis*), Bolivian river dolphin (*Inia boliviensis*), and Franciscana dolphin (*Pontoporia blainvillei*) from Brazil (Figure 1c).

The STs described in this study were submitted to GenBank under the following accession numbers: alphaherpesvirus DPOL nucleotide STs (PX761442-PX761446), gammaherpesvirus DPOL STs (PX761447-PX761449), and gammaherpesvirus gB STs (PX761450-PX761453).

### 3.3. Gross and Histopathological Findings

We analyzed necropsy records of five of the herpesvirus-positive mysticetes in a fresh to moderate decomposed state (code 2). All fresh individuals were identified as humpback whales. A macroscopic examination of the remaining herpesvirus-positive animals was precluded by advanced autolysis. A detailed description of their gross pathological findings is provided in Table S2.

The body condition score was classified into cachectic, poor and good categories. Two of the five herpesvirus-positive individuals presented poor body condition (Mn 1430 and Mn 1052), while three were classified as cachectic (Mn 804, Mn 1081, and Mn 785). On external examination, the presence of foamy material at the respiratory orifices was noted in two individuals (Mn 804 and Mn 1081, Figure S1).

Mild to moderate pulmonary distension was observed in all evaluated individuals, and four of them also exhibited mild to moderate pulmonary edema (Figure S2). The individual Mn 1430 presented omphalitis and fibrinous celomitis. Individual Mn 1052 exhibited cardiac lesions characterized by raised, whitish plaques at the apical regions of the epicardium, endocardium, and cardiac valves - findings consistent with multifocal to coalescing pericarditis (Figure S2). Additionally, one individual (Mn 785) exhibited ectoparasite infestation, multifocal cutaneous lesions of varying morphology, and a gastric mucosal ulcer measuring approximately 6 cm in length, as well as whitish nodules of approximately 1 cm in diameter located in the intestinal submucosa and in the testis (Figure S3). Histopathological examination was precluded by autolytic changes in all positive animals.

## 4. Discussion

To date, this study represents the largest herpesvirus molecular screening conducted in baleen whales of the Southern Hemisphere. Previous reports of herpesviruses in cetaceans were mainly in odontocetes, while in mysticetes they have been largely restricted to isolated cases with no systematic investigations [22,23,25]. The exceptions were the studies by Sacristán et al. [24], which reported a single positive case among 18 humpback whales (5.6%), based mainly in lung and liver samples, and Costa et al. [27], which detected alphaherpesvirus in 10.2% (5/49) of blow samples and gammaherpesvirus in 3.5% (1/29) of the evaluated skin biopsies.

The humpback whale was the most represented species in this research, with 46 individuals evaluated and a herpesvirus detection rate of 23.9%. This rate is apparently lower when compared to previous herpesvirus reports in cetaceans in Brazil with similar sample sizes (e.g., 59.1% (13/22) in Bolivian river dolphins and 51.9% (14/27) in franciscana dolphins) [18], and in countries such as Spain (80% of the striped dolphins *Stenella coeruleoalba*, 28/35) [34]. Other species, like Guiana dolphins (*Sotalia guianensis*) of Brazil, presented a lower detection rate (5.1%, 3/59) [24]. These differences in detection rates likely reflect a combination of biological, ecological, and methodological factors, including sampling design and tissue type. Therefore, direct comparisons of detection rates across species should be interpreted with caution.

This is the second study reporting the presence of herpesviruses in humpback whales of Brazil [24], confirming the occurrence of this viral family and suggesting that the infection may be endemic. Of note, both alphaherpesviruses and gammaherpesviruses were detected, consistent with previous observations in cetaceans [17,18,21,22,25,35], and indicating co-circulation of distinct herpesviral subfamilies in mysticetes. Alphaherpesvirus infections were detected exclusively in humpback whales, whereas gammaherpesviruses were detected in humpback, Bryde’s, and minke whales. To the authors’ knowledge, this is the first report of herpesvirus in Bryde’s whales worldwide, likely broadening the known herpesvirus host range in cetaceans. Furthermore, this constitutes the first detection of a gammaherpesvirus in common minke whales in the southern Atlantic, previously described in the Mediterranean Sea and northeastern Atlantic [22,23] and the second detection of *Gammaherpesvirinae* in humpback whales [27].

Herpesviruses were mainly detected in calves, with 20% positivity in this age class. Given that Brazil is an important breeding area for several mysticetes - especially humpback whales and southern right whales [10,36] - stranded calves are commonly recorded along the coast. Causes of mortality at this age class include fetal stress syndrome, infectious diseases, and trauma [10]. The high detection rate of herpesviruses observed in individuals with such short lifespans (i.e., days to a few weeks) suggests their vertical transmission in whales, which is also suggested for another cetacean species, like the franciscana dolphin [18], and similar to the vertical transmission reported in domestic species and even in humans [37,38]. Alternative routes of infection, including environmental exposure or horizontal postnatal infection, cannot be excluded based on the available data. In calves, intrauterine and colostrum transmissions of herpesviruses can cause neonatal disease and abortion, even in these agents’ natural hosts, highlighting the potential impact in this age class [39].

The molecular detection of herpesvirus DNA does not necessarily indicate active viral replication, as latency is a well-established feature of herpesviruses. However, in three calves and one juvenile, herpesvirus DNA was detected in multiple tissues, suggesting active and potentially systemic infection. Despite viral detection, a gross examination in positive individuals in fresh to mildly decomposed state revealed no lesions attributable to herpesvirus infection, and histopathological evaluation was precluded by autolysis. Field necropsies of mysticetes are limited by their large body size, onceeven in well-preserved carcasses, they are time-consuming, and autolysis may progress before internal organs can be accessed, compromising sample suitability for histopathology. Therefore, it was not possible to determine the presence of alphaherpesvirus- and gammaherpesvirus-associated lesions. Although these viruses can be pathogenic in odontocete cetaceans [17,18,20,21], knowledge on their impact on mysticetes is still limited. To date, an alphaherpesvirus was reported in a whitish skin lesion of a humpback whale with proliferative dermatitis—although causality was not established [25], whereas a gammaherpesvirus was detected in a common minke whale with meningoencephalitis [23]. Additionally, the lack of quantitative assays precluded the assessment of viral load, limiting the inference of infection dynamics. Regarding sex, no significant differences were observed, with seven positive females and six positive males, although the limited sample size precludes robust conclusions.

Sequence analyses confirmed the presence of *Alphaherpesvirinae* and *Gammaherpesvirinae* subfamilies in whales of Brazil. All whale alphaherpesviruses clustered together, forming a distinct lineage, while the gammaherpesviruses likely belong to the genus *Rhadinovirus*, as reported for herpesvirus detected in riverine cetaceans, like Bolivian river dolphins and franciscana dolphins [18], and in Atlantic spotted dolphins [24]. Our findings indicate that cetaceans are infected by at least two gammaherpesvirus genera, *Rhadinovirus* and *Bossavirus*,with the latter being previously reported in odontocetes.

Notably, some of the alphaherpesvirus sequence types detected in humpback whales were highly similar or even identical to those reported in the same species in Norway, Iceland and Cape Verde. Humpback whales migrating to Brazil during austral winter for breeding belong to the Southern Hemisphere breeding stock A and feed during the austral summer in Antarctic waters. In contrast, North Atlantic humpback whales feed during the boreal summer in high-latitude areas (e.g., Norway, Iceland) and breed mainly in Northern Hemisphere tropical regions, such as Cape Verde. There is no evidence of continuous genetic exchange between Northern and Southern Hemisphere humpback whale populations. Thus, it is possible that alphaherpesviruses infected ancestral humpback whale populations and have been maintained independently within these distinct stocks. Further studies, including additional humpback whale breeding stocks and complete genome sequencing, are required to test this hypothesis.

The gammaherpesvirus DPOL sequences from humpback whales were identical to each other and showed low similarity with the closest ones available in public databases. The obtained DPOL gammaherpesvirus sequences from the common minke whale and Bryde’s whale were similar to each other, which may be related to the fact that the DPOL gene is highly conserved, and because both species belong to the same genus, Balaenoptera. The gammaherpesvirus gB sequences identified in this study presented low nt identities to the closest ones available at GenBank, likely due to the lack of previous herpesviruses mysticete gB sequences.

## 5. Conclusions

Overall, this study reports the molecular detection and characterization of herpesviruses in 13 mysticetes stranded along the Brazilian coast, including the first molecular detection of herpesviruses in Bryde’s whales. Additionally, it expands current knowledge on gammaherpesviruses circulating in common minke whales. The detected whale gammaherpesviruses were classified into the genus *Rhadinovirus*. The high detection rate of herpesvirus DNA observed in humpback whale calves suggests vertical transmission in mysticetes. Overall, our findings improve the understanding of herpesvirus diversity, host range, and distribution in mysticetes, and provide baseline data for future epidemiological and conservation-focused studies. Further studies are required to elucidate the impact of herpesviruses on mysticete health, reproduction, and conservation. Accordingly, we recommend incorporating herpesvirus screening into routine health assessments of both stranded and free-ranging whales.

## Figures and Tables

**Table 1:** Biological and epidemiological data of the mysticetes stranded along the Brazilian coast that tested positive for herpesvirus (HV) using PCR protocols to partially amplify the DNA polymerase (DPOL) and glycoprotein B genes (gB).

ID	Species	Region/Location	Year of Stranding	Age Class	Sex ^1^	HV DPOL	HV gB
MM 589	*Balaenoptera brydei*	South	2014	Adult	M	**Positive**	Negative
BA 1681	*Balaenoptera acutorostrata*	South	2021	Calf	F	**Positive**	**Positive**
Mn 785	*Megaptera novaeangliae*	Northeastern	2017	Juvenile	M	**Positive**	**Positive**
Mn 804	*Megaptera novaeangliae*	Northeastern	2017	Calf	M	**Positive**	Negative
Mn 1052	*Megaptera novaeangliae*	Southeastern	2019	Calf	F	**Positive**	Negative
Mn 1054	*Megaptera novaeangliae*	Northeastern	2019	Calf	F	**Positive**	**Positive**
Mn 1081	*Megaptera novaeangliae*	Northeastern	2020	Calf	M	**Positive**	**Positive**
Mn 1430	*Megaptera novaeangliae*	Northeastern	2022	Calf	F	**Positive**	**Positive**
Mn 1608	*Megaptera novaeangliae*	South	2021	Juvenile	F	**Positive**	**Positive**
Mn 1617	*Megaptera novaeangliae*	South	2021	Juvenile	F	**Positive**	Negative
Mn 1619	*Megaptera novaeangliae*	South	2021	Juvenile	F	**Positive**	Negative
Mn 1642	*Megaptera novaeangliae*	South	2021	Juvenile	M	**Positive**	Negative
Mn 1643	*Megaptera novaeangliae*	South	2021	Juvenile	M	**Positive**	Negative

^1^ M = male; F = female; ND = not determined.

**Table 2:** Molecular results and sequence identity analyses of the whales positive for alphaherpesvirus (α-HV) and gammaherpesvirus (γ-HV) detected in this study. Identical sequence types share the same accession number across hosts and/or tissues shown herein.

ID	Species	Positive Organs/ Accession Number	HV Detected	Nucleotide Identity DNA Polymerase	Amino Acid Identity DNA Polymerase	Nucleotide Identity Glycoprotein B	Amino Acid Identity Glycoprotein B
MM 589	*Balaenoptera brydei*	DPOL: bladder PX761447	γ-HV	99.4% to a γ-HV sequence detected in a common minke whale of Spain (KP995688)	100% to a γ-HV sequence from a common minke whale of Spain (KP995688)	Not determined	Not determined
BA 1681	*Balaenoptera acutorostrata*	DPOL: lymph node PX761448 gB: lymph node and kidney PX761453	γ-HV	95.8% to a γ-HV sequence detected in a common minke whale of Spain (KP995688)	94.5% to a γ-HV sequence from a common minke whale of Spain (KP995688)	81.3% to γ-HV sequences from Atlantic spotted dolphins (OQ926585, OQ926586)	84.5% to γ-HV sequences from Atlantic spotted dolphins (OQ926585, OQ926586)
Mn 785	*Megaptera novaeangliae*	DPOL: skin PX761449 gB: lymph node PX761450	γ-HV	81.4% to a γ-HV sequence from a common minke whale of Spain (KP995688)	85.5% to a γ-HV sequence from a common minke whale of Spain (KP995688)	79.7% to γ-HVs from Atlantic spotted dolphins (*Stenella frontalis*, OQ926585, OQ926586) and Bolivian river dolphin (*Inia boliviensis*, MZ209258)	Identical to γ-HV sequences from Mn 1081 and Mn 1608. The aa identity percentages are the same as those described for case Mn 1081
Mn 804	*Megaptera novaeangliae*	DPOL: lung PX761449	γ-HV			Not determined	Not determined
Mn 1052	*Megaptera novaeangliae*	DPOL: lymph node PX761449	γ-HV			Not determined	Not determined
Mn 1054	*Megaptera novaeangliae*	DPOL: kidney PX761449 gB: kidney PX761451	γ-HV			79.2% to γ-HVs detected in Atlantic spotted dolphins (*Stenella frontalis*, OQ926585, OQ926586) and Bolivian river dolphin (*Inia boliviensis*, MZ209258)	84.5% to γ-HVs detected in Atlantic spotted dolphins (*Stenella frontalis*, OQ926585, OQ926586) and Bolivian river dolphin (*Inia boliviensis*, MZ209258)
Mn 1081	*Megaptera novaeangliae*	DPOL: lung PX761449 gB: brain, lymph node and lung PX761452	γ-HV			79.4% to γ-HVs from Atlantic spotted dolphins (*Stenella frontalis*, OQ926585, OQ926586) and Bolivian river dolphin (*Inia boliviensis*, MZ209258)	85.2% to γ-HVs from Atlantic spotted dolphins (*Stenella frontalis*, OQ926585, OQ926586) and Bolivian river dolphin (*Inia boliviensis*, MZ209258)
Mn 1430	*Megaptera novaeangliae*	DPOL: kidney and heart PX761449 gB: kidney Not available	γ-HV			γ-HV sequence with poor quality for identity analysis	γ-HV sequence with poor quality for identity analysis
Mn 1608	*Megaptera novaeangliae*	DPOL: kidney and skin PX761449 gB: skin PX761452	α-HV and γ-HV	Two sequences: - α-HV with 95.3% to detected in a humpback whale from Norway (OQ561785) -γ-HV with 81.4% to a sequence detected in the common minke whale of Spain (KP995688)	Two sequences: - 97.2% to an α-HV sequence from a humpback whale from Norway (OQ561785) - 85.5% to a γ-HV sequence detected in the common minke whale of Spain (KP995688)	79.4% to γ-HV sequences from Atlantic spotted dolphins (*Stenella frontalis*, OQ926585, OQ926586) and Bolivian river dolphin (*Inia boliviensis*, MZ209258)	Identical to γ-HV sequences from Mn 1081 and Mn 705. The aa identity percentages are the same as those described for case Mn 1081
Mn 1617	*Megaptera novaeangliae*	DPOL: lung PX761442	α-HV	100% to a sequence from humpback whales of Brazil (OQ533671), Iceland (PP857848), Norway (PP857844) and Cape Verde (PP857846)	100% to a sequence from humpback whales of Brazil (OQ533671), Iceland (PP857848), Cape Verde (PP857846) and Norway (PP857843, PP857844, PP857845)	Not determined	Not determined
Mn 1619	*Megaptera novaeangliae*	DPOL: lung PX761443	α-HV	99.4% to a sequence detected in humpback whales from Brazil (OQ533671), Iceland (PP857848), Norway (PP857844) and Cape Verde (PP857846)	100% to a sequence from humpback whales from Brazil (OQ533671), Iceland (PP857848), Cape Verde (PP857846) and Norway (PP857843, PP857844, PP857845)	Not determined	Not determined
Mn 1642	*Megaptera novaeangliae*	DPOL: lung PX761444	α-HV	100% to a sequence from a humpback whale from Norway (PP857845)	100% to a sequence detected in humpback whales from Brazil (OQ533671), Iceland (PP857848), Cape Verde (PP857846) and Norway (PP857843, PP857844, PP857845)	Not determined	Not determined
Mn 1643	*Megaptera novaeangliae*	DPOL: kidney PX761445	α-HV	99.4% to a sequence detected in a humpback whale from Norway (PP857845)	98.2% to a sequence detected in humpback whales from Brazil (OQ533671), Iceland (PP857848), Cape Verde (PP857846) and Norway (PP857843, PP857844, PP857845)	Not determined	Not determined

## Data Availability

All data generated or analyzed during this research are included in the manuscript. The DNA polymerase nucleotide sequences obtained in this research were submitted to GenBank under accession numbers PX761442-PX761453.

## Supplementary Materials

The following supporting information can be downloaded at https://www.mdpi.com/article/10.3390/v18060612/s1, Table S1. Biological and epidemiological data of the mysticetes stranded along the Brazilian coast and tested for herpesvirus using PCR protocols to partially amplify the DNA polymerase (DPOL) and glycoprotein B genes (gB). Table S2. Main pathological findings in fresh herpesvirus-positive mysticetes stranded on the Brazilian coast. Figure S1. (a) MN 1081—Mild to moderate presence of foam at the right respiratory orifice. (b) MN 804—Moderate amount of serosanguineous foam at the respiratory orifices. Figure S2. (a) MN 1430—Pulmonary enlargement evidenced by rib impressions on the pleural surface. Sectioning revealed marked mild to moderate congestion and edema. (b) MN 804—Upon sectioning, the lung parenchyma was diffusely dark red to blackish, with mild to moderate amounts of serosanguineous foamy fluid. (c) MN 1052—Multifocal to coalescing raised whitish plaques adhered to the apical region of the pericardium, consistent with multifocal to coalescing fibrinous pericarditis. (d) MN 1430—Umbilicus cleft contained three coalescing, verrucous nodules, suggestive of omphalitis. Figure S3. (a) and (b) MN 785—Cutaneous lesions involving approximately 30–40% of the total dorsolateral body surface of the animal, including moderate epidermal thickening on the dorsal region, conferring a crusted appearance, and discrete to moderate multifocal to coalescing circular whitish/hypopigmented areas in dorsolateral regions extending from the dorsal to the caudal fin and affecting the pectoral fins. (c) Focally expansive cutaneous lesion with predominantly reddish to brownish crusted coloration, with numerous ectoparasites associated with areas of erosion inferior to the dorsal fin on the right lateral region. (d) Gastric chamber II containing a moderate focal ulcer measuring approximately 6 cm in diameter in the gastric mucosa. (e) The testicular serosal layer shows a focal, oval, whitish nodule measuring approximately 1 cm long.
