# Supplementary material for: Herpesvirus Diversity in Stranded Mysticetes of Brazil, Southwestern Atlantic
Source: Viruses. 2026 May 27;18(6):612. doi: 10.3390/v18060612 (PMC13307567; doi:10.3390/v18060612)
Supplement: Supplementary file 1 [file viruses-18-00612-s001.zip › viruses-4271845-supplementary.pdf]

**Table S1.** Biological and epidemiological data of the mysticetes stranded along the Brazilian coast and tested for herpesvirus using PCR protocols to partially amplify the DNA polymerase (DPOL) and glycoprotein B genes (gB).

| Number | Original ID | Species | Age class | Sex[1] | Year of stranding | Region | HV DPOL | HV gB |
|---|---|---|---|---|---|---|---|---|
| 1 | Mn 804 | *Megaptera novaeangliae* | Calf | M | 2017 | Northeastern | **Positive** | Negative |
| 2 | Mn 801 | *Megaptera novaeangliae* | Calf | F | 2017 | Northeastern | Negative | Negative |
| 3 | Mn 845 | *Megaptera novaeangliae* | Calf | M | 2017 | Northeastern | Negative | Negative |
| 4 | Mn 835 | *Megaptera novaeangliae* | Adult | M | 2017 | Northeastern | Negative | Negative |
| 5 | Mn 828 | *Megaptera novaeangliae* | Calf | F | 2017 | Southeastern | Negative | Negative |
| 6 | Mn 863 | *Megaptera novaeangliae* | Calf | F | 2017 | Northeastern | Negative | Negative |
| 7 | Mn 855 | *Megaptera novaeangliae* | Calf | M | 2017 | Northeastern | Negative | Negative |
| 8 | Mn 1081 | *Megaptera novaeangliae* | Calf | M | 2020 | Northeastern | **Positive** | **Positive** |
| 9 | Mn 1054 | *Megaptera novaeangliae* | Calf | F | 2019 | Northeastern | **Positive** | **Positive** |
| 10 | Mn 785 | *Megaptera novaeangliae* | Juvenile | M | 2017 | Northeastern | **Positive** | **Positive** |
| 11 | Mn 969 | *Megaptera novaeangliae* | Adult | M | 2018 | Southeastern | Negative | Negative |

| Number | Original ID | Species | Age class | Sex[1] | Year of stranding | Region | HV DPOL | HV gB |
|---|---|---|---|---|---|---|---|---|
| 12 | Mn 815 | *Megaptera novaeangliae* | Juvenile | M | 2017 | Southeastern | Negative | Negative |
| 13 | Mn 798 | *Megaptera novaeangliae* | ND | ND | 2017 | Northeastern | Negative | Negative |
| 14 | Mn 803 | *Megaptera novaeangliae* | Calf | F | 2017 | Southeastern | Negative | Negative |
| 15 | Mn 518 | *Megaptera novaeangliae* | Calf | F | 2012 | Northeastern | Negative | Negative |
| 16 | Mn 844 | *Megaptera novaeangliae* | Calf | ND | 2017 | Northeastern | Negative | Negative |
| 17 | Mn 856 | *Megaptera novaeangliae* | Calf | F | 2017 | Northeastern | Negative | Negative |
| 18 | MM 589 | *Balaenoptera brydei* | Adult | M | 2014 | South | **Positive** | Negative |
| 29 | MM 569 | *Balaenoptera brydei* | Juvenile | M | 2018 | Southeastern | Negative | Negative |
| 20 | Mn 1430 | Megaptera novaeangliae | Calf | F | 2022 | Northeastern | **Positive** | **Positive** |
| 21 | Mn 972 | *Megaptera novaeangliae* | Juvenile | M | 2018 | Northeastern | Negative | Negative |
| 22 | BE 814 | *Balaenoptera brydei* | Calf | F | 2017 | Northeastern | Negative | Negative |
| 23 | Mn 1619 | *Megaptera novaeangliae* | Juvenile | F | 2021 | South | **Positive** | Negative |
| 24 | Mn 1024 | *Megaptera novaeangliae* | Calf | M | 2019 | Southeastern | Negative | Negative |
| 25 | Mn 1418 | *Megaptera novaeangliae* | Calf | M | 2022 | Northeastern | Negative | Negative |

| Number | Original ID | Species | Age class | Sex[1] | Year of stranding | Region | HV DPOL | HV gB |
|---|---|---|---|---|---|---|---|---|
| 26 | Mn 944 | *Megaptera novaeangliae* | Adult | M | 2018 | Northeastern | Negative | Negative |
| 27 | Mn 916 | *Megaptera novaeangliae* | Adult | M | 2018 | Northeastern | Negative | Negative |
| 28 | Mn 982 | *Megaptera novaeangliae* | Calf | M | 2018 | Northeastern | Negative | Negative |
| 29 | Mn 826 | *Megaptera novaeangliae* | Calf | F | 2017 | Southeastern | Negative | Negative |
| 30 | Mn 593 | *Megaptera novaeangliae* | Calf | M | 2013 | Northeastern | Negative | Negative |
| 31 | Mn 1440 | *Megaptera novaeangliae* | Calf | M | 2022 | Northeastern | Negative | Negative |
| 32 | Mn 1052 | *Megaptera novaeangliae* | Calf | F | 2019 | Southeastern | **Positive** | Negative |
| 33 | Mn 301 | *Megaptera novaeangliae* | Calf | M | 2008 | Southeastern | Negative | Negative |
| 34 | Mn 345 | *Megaptera novaeangliae* | Calf | F | 2009 | Southeastern | Negative | Negative |
| 35 | Mn 333 | *Megaptera novaeangliae* | Juvenile | F | 2009 | Northeastern | Negative | Negative |
| 36 | Mn 1482 | *Megaptera novaeangliae* | Calf | M | 2022 | Southeastern | Negative | Negative |
| 37 | Mn 975 | *Megaptera novaeangliae* | Calf | M | 2018 | Northeastern | Negative | Negative |
| 38 | Mn 943 | *Megaptera novaeangliae* | Calf | M | 2018 | Northeastern | Negative | Negative |
| 39 | Mn 1405 | *Megaptera novaeangliae* | Juvenile | M | 2022 | Northeastern | Negative | Negative |

| Number | Original ID | Species | Age class | Sex[1] | Year of stranding | Region | HV DPOL | HV gB |
|---|---|---|---|---|---|---|---|---|
| 40 | Mn 1422 | *Megaptera novaeangliae* | Calf | M | 2022 | Northeastern | Negative | Negative |
| 41 | Mn 1479 | *Megaptera novaeangliae* | Adult | F | 2022 | Southeastern | Negative | Negative |
| 42 | Mn 1408 | *Megaptera novaeangliae* | Juvenile | F | 2022 | Northeastern | Negative | Negative |
| 43 | BA 1681 | *Balaenoptera acutorostrata* | Calf | F | 2021 | South | **Positive** | **Positive** |
| 44 | Mn 1598 | *Megaptera novaeangliae* | Juvenile | M | 2021 | South | Negative | Negative |
| 45 | Mn 1643 | *Megaptera novaeangliae* | Juvenile | M | 2021 | South | **Positive** | Negative |
| 46 | EA 1618 | *Eubalaena australis* | Calf | F | 2021 | South | Negative | Negative |
| 47 | Mn 1658 | *Megaptera novaeangliae* | Juvenile | M | 2021 | South | Negative | Negative |
| 48 | Mn 1642 | *Megaptera novaeangliae* | Juvenile | M | 2021 | South | **Positive** | Negative |
| 49 | Mn 1617 | *Megaptera novaeangliae* | Juvenile | F | 2021 | South | **Positive** | Negative |
| 50 | Mn 1608 | *Megaptera novaeangliae* | Juvenile | F | 2021 | South | **Positive** | **Positive** |
| 51 | Mn 578 | *Megaptera novaeangliae* | Calf | M | 2013 | Southeastern | Negative | Negative |

[1]M = male, F = female, ND = not described.

**Table S2.** Main pathological findings in fresh herpesvirus-positive mysticetes stranded on the Brazilian coast.

| Number | ID | Species | Gross findings |
|---|---|---|---|
| 1 | 804 | *Megaptera novaeangliae* | **Body condition score:** Cachectic.<br>**External examination**: Moderate serosanguineous foam at the respiratory orifices. Shark bite associated with laceration and moderate hemorrhage on tongue.<br>**Subcutaneous:** Multifocal to coalescing petechiae, effusions and mild to moderate ecchymoses. Fat atrophy on blubber.<br>**Lungs:** Mild to moderate presence of serosanguineous foamy content.<br>**Heart:** Marked diffuse serous fat atrophy.<br>**Intestinal tract, liver, spleen, kidneys and urinary bladder:** NSFO. |
| 8 | 1081 | *Megaptera novaeangliae* | **Body condition score:** Cachectic.<br>**External examination:** Mild to moderate presence of foam at the right respiratory orifice. Mild multifocal abrasion along the cranial margi of the pectoral fins. Mild focal ecchymosis on ventral region of the lower lip. Plolapsed penis and presence of fecal material at the anal opening.<br>Left eye presenting moderate hyphema.<br>**Subcutaneous tissue:** Serous coloration.<br>**Muscle and serosal surfaces:** Diffuse red-withish coloration.<br>**Lungs**: Mild pulmonary enlargement.<br>**Bronchi and trachea:** Moderate foamy content.<br>**Heart**: Diverticulum measuring approximately 5-6 cm in the greatest axis adhered to the atrial external surface.<br>**Liver, spleen and kidneys:** Mild to moderate congestion.<br>**Gastrointestinal tract and urinary bladder:** NSFO. |
| 10 | 785 | *Megaptera novaeangliae* | **Body condition score:** Cachectic.<br>**External examination:** Cutaneous lesions affected approximately 30–40% of the dorsolateral body surface. These included mild to moderate multifocal abrasions on the left lateral region and along the margins of the left pectoral fin, with exposure of hemorrhagic subcutaneous tissue. Moderate epidermal thickening on the dorsal |

| | | | |
|---|---|---|---|
| | | | surface, along with multifocal to coalescing mild to moderate hypopigmented circular areas in the dorsolateral regions, extending from the dorsal fin to the caudal fin and involving the pectoral fins. On the right lateral aspect, ventral to the dorsal fin, a focally expansive area with predominantly reddish to brownish crusted coloration was noted, associated with ectoparasites and areas of cutaneous erosion. Mild multifocal cookiecutter shark bite marks were present. In addition, there was a disseminated infestation of *Cyamidae* ectoparasites and multifocal presence of *Coronula diadema* on the head and pectoral fins.<br>**Subcutaneous:** Mild to moderate ecchymosis.<br>**Lungs:** Moderate enlargement and mild pulmonary edema.<br>**Bronchi and trachea**: Mild to moderate serosanguineous foam.<br>**Digestive tract:** Moderate enteritis; multifocal nodules on intestinal submucosa, measuring approximately 1cm diameter; on cut surface, were homogeneously yellow, without content exsudation. Focal, moderate gastric mucosal ulcer in stomach chamber II.<br>**Liver:** Capsule showed reticular thickening with opaque grayinsh-white appearance. Non-homogeneous wine-red coloration.<br>**Spleen:** Mild congestion. |
| 20 | 1430 | *Megaptera novaeangliae* | **Body condition score:** Poor.<br>**External examination:** Regular linear , polygonal impressions extending from the mid-dorsal region of the head to the caudal peduncle, involving the right lateral region and the right pectoral fin, consistent with fishing net entanglement. Shark bite on the left pectoral fin. Umbilicus showed omphalitis.<br>**Subcutaneous and muscular tissue**: Diffusely pale, with reddish-withish to pink coloration.<br>**Lungs**: Diffuse pulmonary enlargement, and mild to moderate congestion and edema.<br>**Gastrointestinal tract:** Moderate fibrinous celomitis.<br>**Urinary bladder:** Mild cystitis.<br>**Heart, liver, spleen, and kidneys:** NSFO. |

| | | | |
|---|---|---|---|
| 32 | 1052 | *Megaptera novaeangliae* | **Body condition score:** Poor.<br>**External examination:** Multiple linear abrasions on the right lateral region of the head, dorsal body surface and dorsal surfaces of pectoral fins. Multiple linear incisions compatible with anthropogenic interaction, located on the right lateral region of the rostrum and dorsal region of the head, approximately 3 to 7 cm in length. Vulture bites in the right periocular region and in the umbilicus.<br>**Subcutaneous tissue:** Diffuse, homogeneous pale.<br>**Lungs:** Moderately distended. Sectioning revealed marked mild to moderate congestion and edema.<br>**Heart:** Mild to moderate congestion; mild multifocal to coalescing pericarditis.<br>**Liver:** Hepatic hematoma.<br>**Kidneys:** Mild to moderate congestion.<br>**Digestive tract, spleen and urinary bladder:** NSFO. |

*NSFO: No significant findings observed

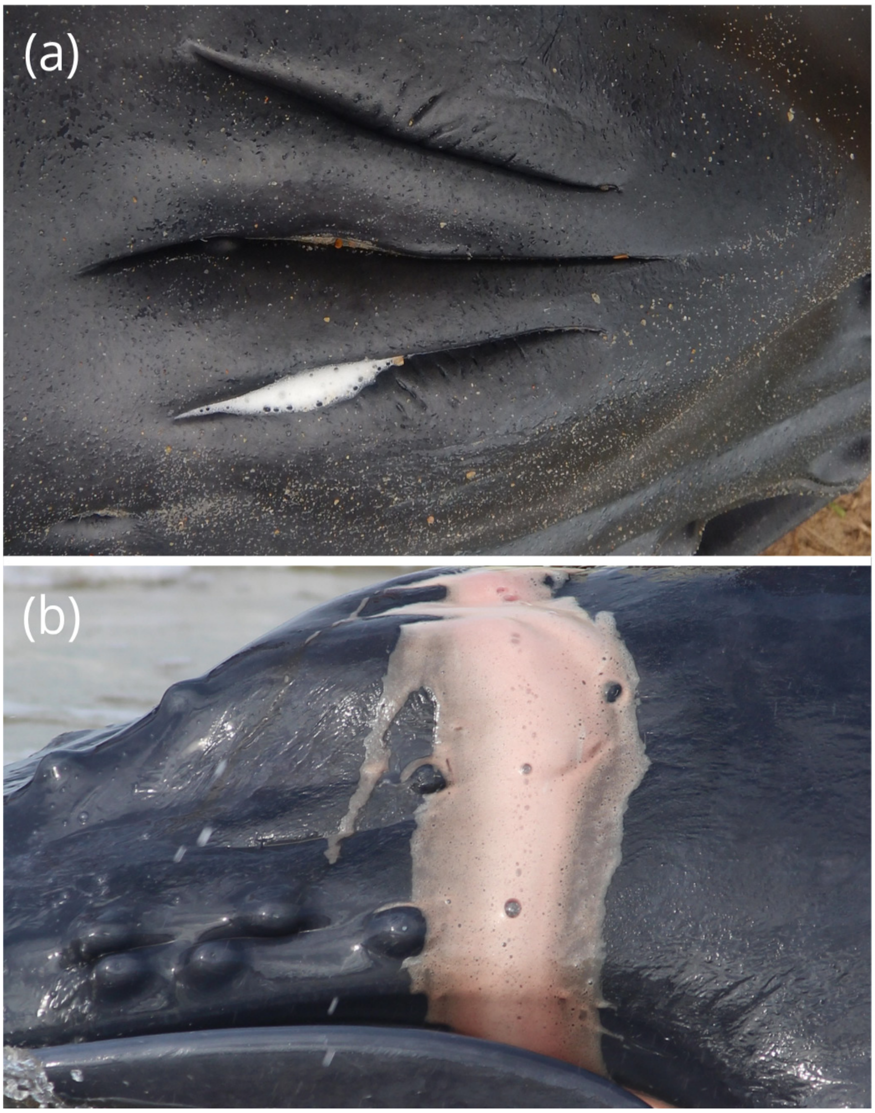


Figure S1. **(a)** MN 1081 - Mild to moderate presence of foam at the right respiratory orifice. **(b)** MN 804 – Moderate amount of serosanguineous foam at the respiratory orifices.

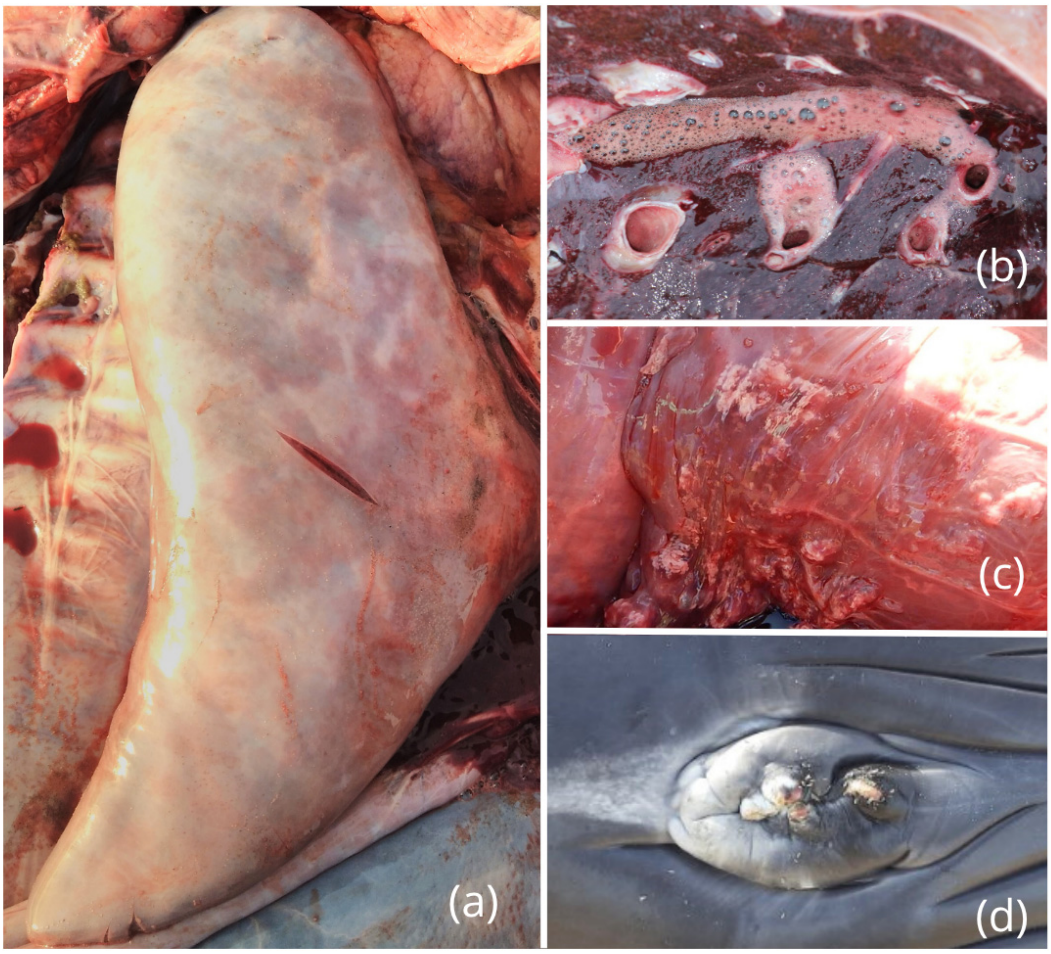


**Figure S2. (a)** MN 1430 - Pulmonary enlargement evidenced by rib impressions on the pleural surface. Sectioning revealed marked mild to moderate congestion and edema. **(b)** MN 804 - On sectioning, the lung parenchyma was diffusely dark red to blackish, with mild to moderate amounts of serosanguineous foamy fluid. **(c)** MN 1052 - Multifocal to coalescing, raised, whitish plaques adhered to the apical region of the pericardium, consistent with multifocal to coalescing fibrinous pericarditis. **(d)** MN 1430 - Umbilicus cleft contained three coalescing, verrucous nodules, suggestive of omphalitis.

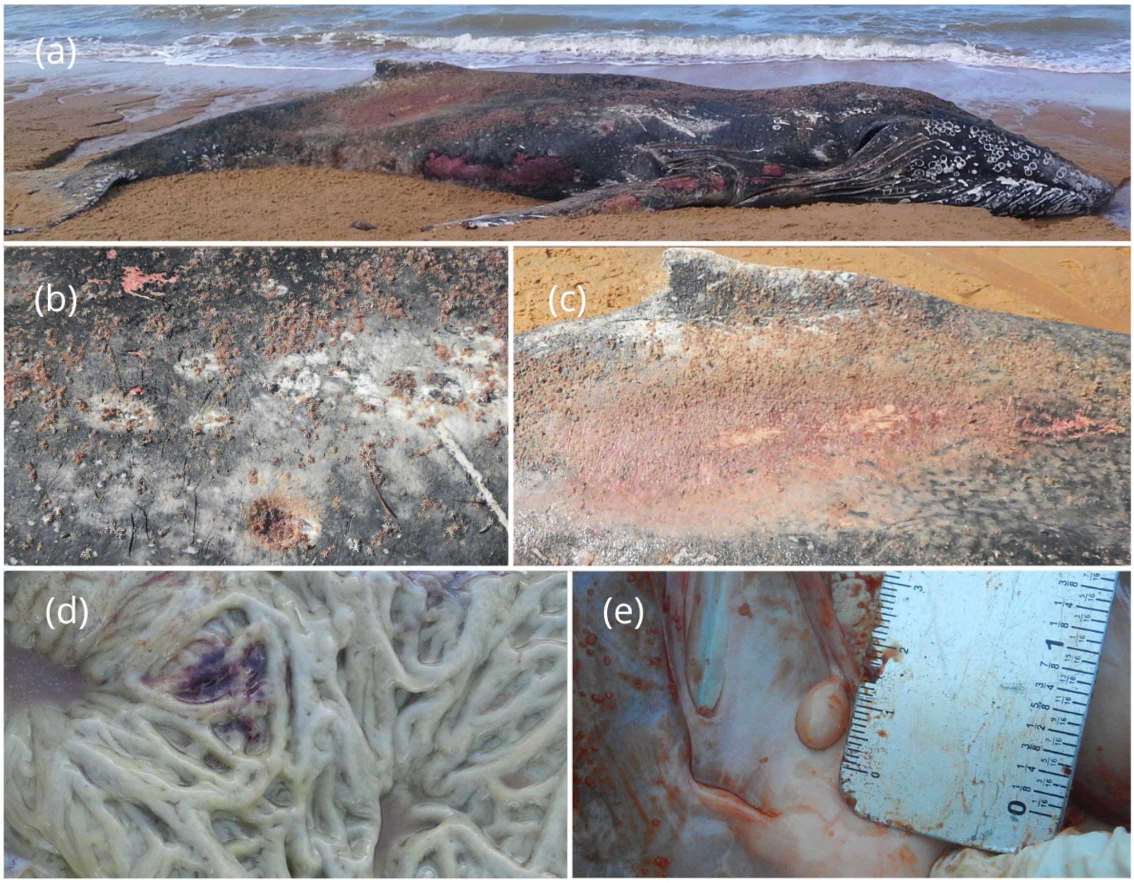


**Figure S3. (a)** and **(b)** MN 785 - Cutaneous lesions involving approximately 30–40% of the total dorsolateral body surface of the animal, including moderate epidermal thickening on the dorsal region, conferring a crusted appearance, and discrete to moderate multifocal to coalescing circular whitish/hypopigmented areas in dorsolateral regions extending from the dorsal to the caudal fin and affecting the pectoral fins. **(c)** Focally expansive cutaneous lesion with predominantly reddish to brownish crusted coloration, with numerous ectoparasites associated with areas of erosion inferior to the dorsal fin on the, on the right lateral region. **(d)** Gastric chamber II containing a moderate focal ulcer measuring approximately 6 cm in diameter in the gastric mucosa. **(e)** The testicular serosal layer shows a focal, oval, whitish nodule measuring approximately 1 cm long.
